# Three-dimensional visualization of dentine occlusion based on FIB-SEM tomography

**DOI:** 10.1038/s41598-023-29155-1

**Published:** 2023-02-08

**Authors:** Xinye Chen, Kaleigh M. Ryan, Deon Hines, Long Pan, Ke Du, Shiyou Xu

**Affiliations:** 1 Colgate Technology Center, Piscataway, NJ 08854 USA; 2 Microsystems Engineering, Rochester Institute of Technology, Rochester, NY 14623 USA; 3 Chemical and Environmental Engineering, University of California Riverside, Riverside, CA 92508 USA; 4 Department of Materials Science and Engineering, Rutgers University, Piscataway, NJ 08854 USA

**Keywords:** Occlusion, Tissues, Imaging techniques

## Abstract

The occlusion of dentinal tubules has become a rapid and effective method for treating dentin hypersensitivity. Accurate evaluation of dentin occlusion is critical to illustrate the efficacy of oral care products and to optimize dental therapy in the clinics, which is limited by the conventional two-dimensional (2-D) characterization methods. Here, we demonstrate the visualization of the dentin occlusion via three-dimensional (3-D) characterization using a focused ion beam-scanning electron microscopy (FIB-SEM) tomography. Using the “Slice and View” approach, the material used for occluding dentin tubules is imaged with a very high-resolution voxel (10 nm × 10 nm × 20 nm) from 2-D SEM images and then reconstructed into a 3-D volume, which presents the mode of action of toothpaste for treating dentin hypersensitivity. Meanwhile, quantitative analysis of the depth of occlusion is successfully obtained. This work validates the feasibility of FIB-SEM tomography in the analysis of dentin occlusion within the complicated networks of dentine tubules at the nanoscale, and provides a novel approach to facilitate the research and development of oral care products.

## Introduction

Dentin hypersensitivity has become a growing oral health concern globally^1^. As reported, adults between the ages of 20 to 50 are frequently diagnosed with dentine hypersensitivity at a prevalence rate of up to 98%^2,3^. In addition, the prevalence distribution of patients with chronic periodontal diseases has been recognized to be above 72%^2,4^. Dentin hypersensitivity is defined as a sharp and localized pain for a short period, typically associated with a stimulus of thermal, osmotic, or chemical source to the exposed dentin tubules^5^, but not responsible for any other tooth defects or diseases^6^. Dentin hypersensitivity is caused by the exposed and open dentin tubules, owing to enamel erosion and gingival recession^7^. The available treatment strategies for dentin hypersensitivity, either at home or in office, can be classified into two groups by the mechanism of actions, which are the obstruction of the neural response to pain stimulus and the occlusion of dentinal tubules^8^. Occluding the open dentin tubules with specific materials could be one of the most principal methods, which can immediately and efficiently reduce dentin hypersensitivity due to the direct interruption of the sensitive mechanisms^9^.

Many models and simulations related to dentin hypersensitivity have been reported previously to simplify the matrix of dentin tubules, such as considering it as a linear non-branched structure^10^. However, the real tubule network features micro- or nano-branching and interconnection^11^. If the tubules sample has been deposited by dentin hypersensitivity treatment, the real sample are much more complicated than the simplified non-branched model^10^. Therefore, it is necessary to apply microscopic approaches to probe the details of occluded dentin tubules in 2-D, and 3-D, to enable more comprehensive evaluation of impacted treatment on the nanoscale components. Various 2-D characterization techniques, such as confocal microscopy^12,13^ and scanning electron microscopy (SEM)^14,15^, have been used to determine the efficacy of targeted material (e.g., desensitized dentifrice for treating dentin hypersensitivity). However, these methods have a relatively low resolution and/or only provide 2-D information, thus a comprehensive image of dentine occlusion is not attainable by these methods. X-ray microtomography is a developed tomographic technique which displayed potential for 3D characterization of dentin tubules^16,17^, but the spatial resolution is in the order of several hundred nanometers. Such spatial resolution would not be able to provide much more details on the nano-branching portion of the tubule network.

A comprehensive visualization of dentinal tubule occlusion in 3-D domain could significantly promote the development for a more effective therapy of dentin hypersensitivity by enabling a better understanding of the relationship between functional materials used for occlusion and the spatial structure of dentin. Currently, focused ion beam-scanning electron microscopy (FIB-SEM) tomography has been well developed and used as a promising analytical approach based on high-resolution 3-D reconstruction techniques^18,19^. This technique allows one to quantitatively explore the ultrastructural properties of colloidal nanoparticles^20,21^ and biological samples^22,23^. FIB-SEM is a very robust technique, featuring the capability of 3-D real-space imaging of up to thousands of cubic microns while offering a very high resolution of down to several nanometers^24^. The precise control of both the focused ions and the electron beam are usually based on an independent operation system, simultaneously allowing the accurate milling process from FIB scanning and the high-resolution images captured from SEM recording^25^.

In this study, we developed a desirable method dependent on a 3-D FIB-SEM tomography to quantitatively visualize the spatial distribution of particle-occluded dentin tubules by a programmed reconstruction of image stacking. The method presented here can generate novel insights into the evaluation of tubule-occluding efficacy. This FIB-SEM collaboration process can eventually provide a high-quality 3-D reconstruction of the specific specimen with a high-resolution voxel of 5 nm × 5 nm × 5 nm, thus extending to more delicate exploration of even a single dentinal tubule with nanostructures. This imaging technique could offer guidance for dentists to determine the performance of applied treatments and it can be utilized in the development of new treatment products for dentin hypersensitivity.

## Results

**Figure 1:**
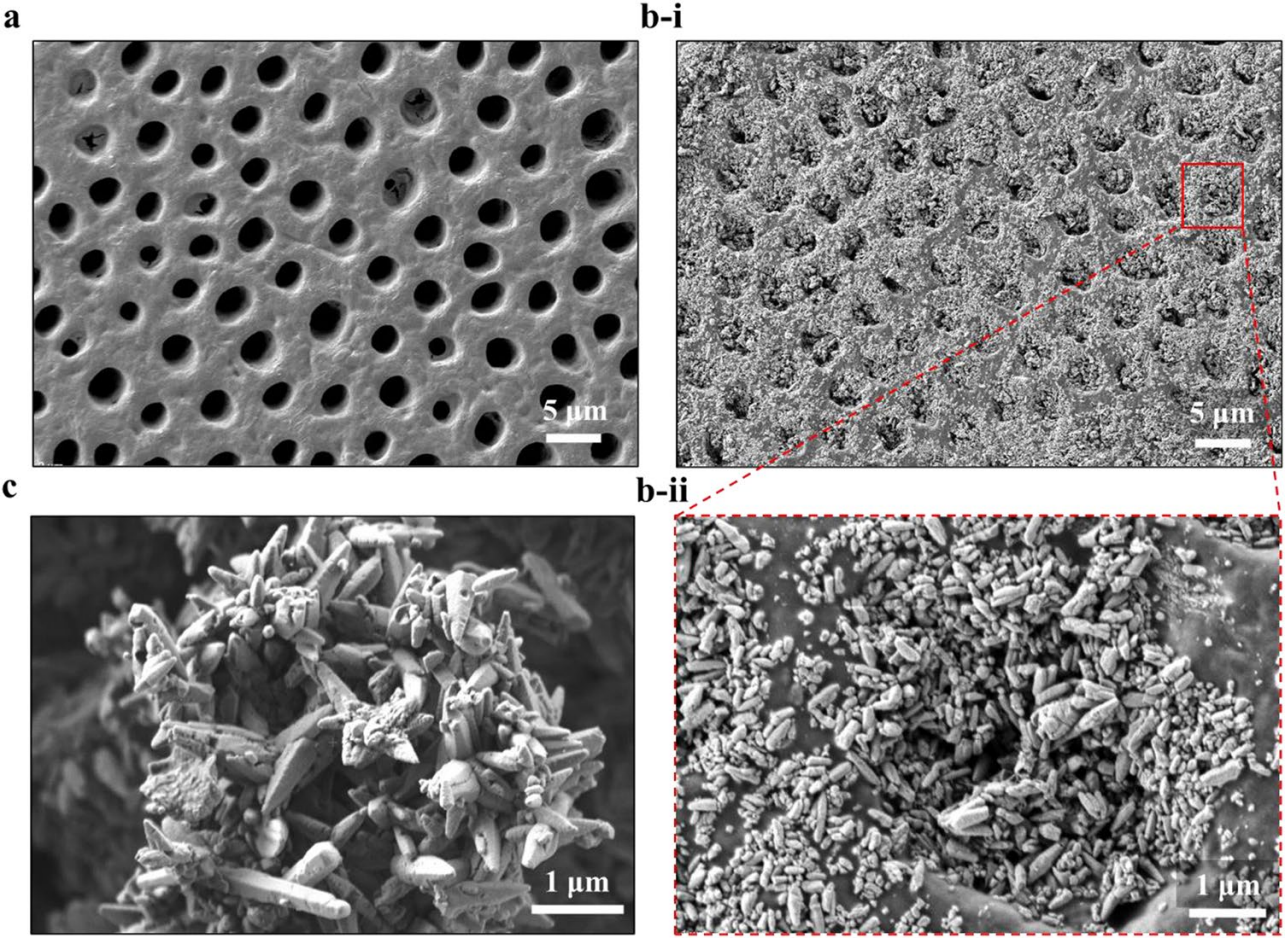
SEM images displaying (**a**) the surface of the cleaned dentinal specimen before applying the testing toothpaste; (**b**) the surface of the treated dentin disc showing occlusion by the test toothpaste under low magnification (**b-i**) and high magnification (**b-ii**); and (**c**) the morphology of raw material used for forming the testing toothpaste.

Figure 1 shows the representative SEM images of the dentinal specimens before and after applying the testing toothpaste, and the comparison of the raw material and the testing toothpaste within the tubules through the morphology of the involved calcium carbonate (CaCO_3_). Figure 1a presents a typical untreated specimen with the cleaned and smoothed surface, where the dentinal tubules are completely open without any smear layer or smear plugs. In Fig. 1b-i, the treated dentin surface exhibits that almost all open tubules are blocked or plugged by the test toothpaste. An individual occluded tubule is enlarged at a higher magnification (Fig. 1b-ii), which is compared to the raw material clearly shown in Fig. 1c. Both materials consisting of CaCO_3_ nanoparticles present a similar morphology, confirming that the test toothpaste successfully attaches to the dentinal specimen surface and occluded the dentinal tubule. The occlusion depth will then be analyzed through cross-sectional images of the tubules.

**Figure 2:**
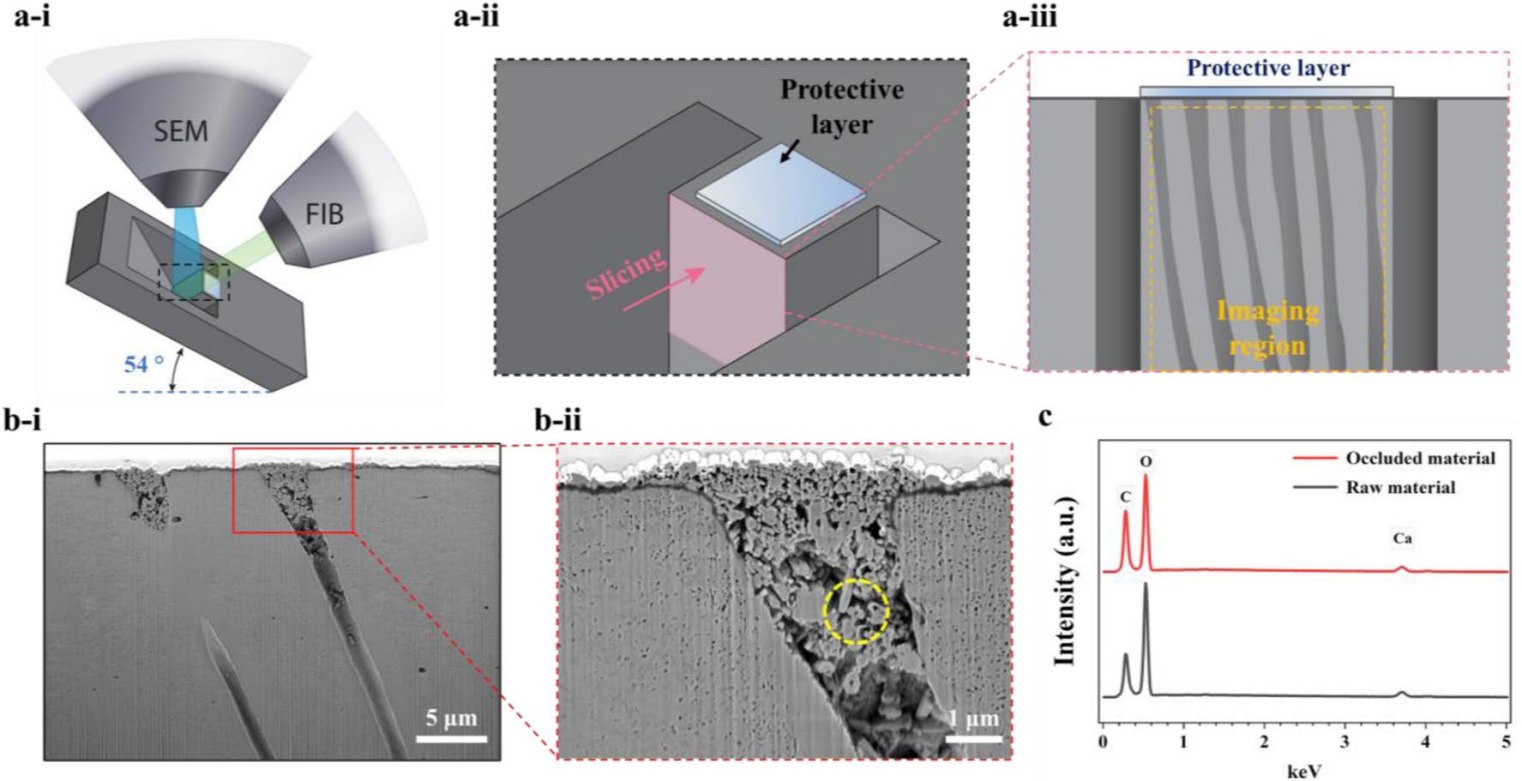
The EDS characterization of occluded tubules using FIB-SEM approach. (**a-i**) The schematic of the specimen setup in the FIB-SEM chamber for data acquisition. (**a-ii**) The illustration of a volume of dentin material prepared for serial FIB slicing. The pink arrow indicates the FIB slicing along the longitudinal section. A thin layer of Pt is deposited before FIB slicing. (**a-iii**) An example of a cross-section presenting the exposed tubules during FIB slicing. (**b**) The cross-sectional view of the dentinal tubule occluded by the testing toothpaste under low magnification (**b-i**) and high magnification (**b-ii**). The yellow dashed circle indicates the location of probing the occluded material within the dentinal tubule via EDS analysis (**c**).

To further characterize the dentin tubule occlusion caused by the raw material in the toothpaste, the dentinal specimen was mounted in the FIB-SEM chamber as shown in Fig. 2a-i. A protective layer of Pt was deposited (thickness: 200 nm). The longitudinal FIB slicing, milling the sample along the length direction of the dentin tubules, was applied (Fig. 2a-ii). This was performed to expose the tubules’ cross-section (Fig. 2a-iii) to analyze the components of the test toothpaste occluded within the tubules as presented in Fig. 2b-i. The high-magnification SEM image displays a well-occluded tubule (Fig. 2b-ii). The EDS spectrum was used to present the typical composition of the occluded testing toothpaste. As shown in Fig. 2c, the particles occluded in the dentin tubules consist of the same elements as the raw material, which are carbon (C), oxygen (O), and calcium (Ca). The EDS spectrum confirms that the material occluded in the dentin tubule is from the major component of the test toothpaste, which is CaCO_3_, and designed for treating dentin hypersensitivity by occlusion.

**Figure 3:**
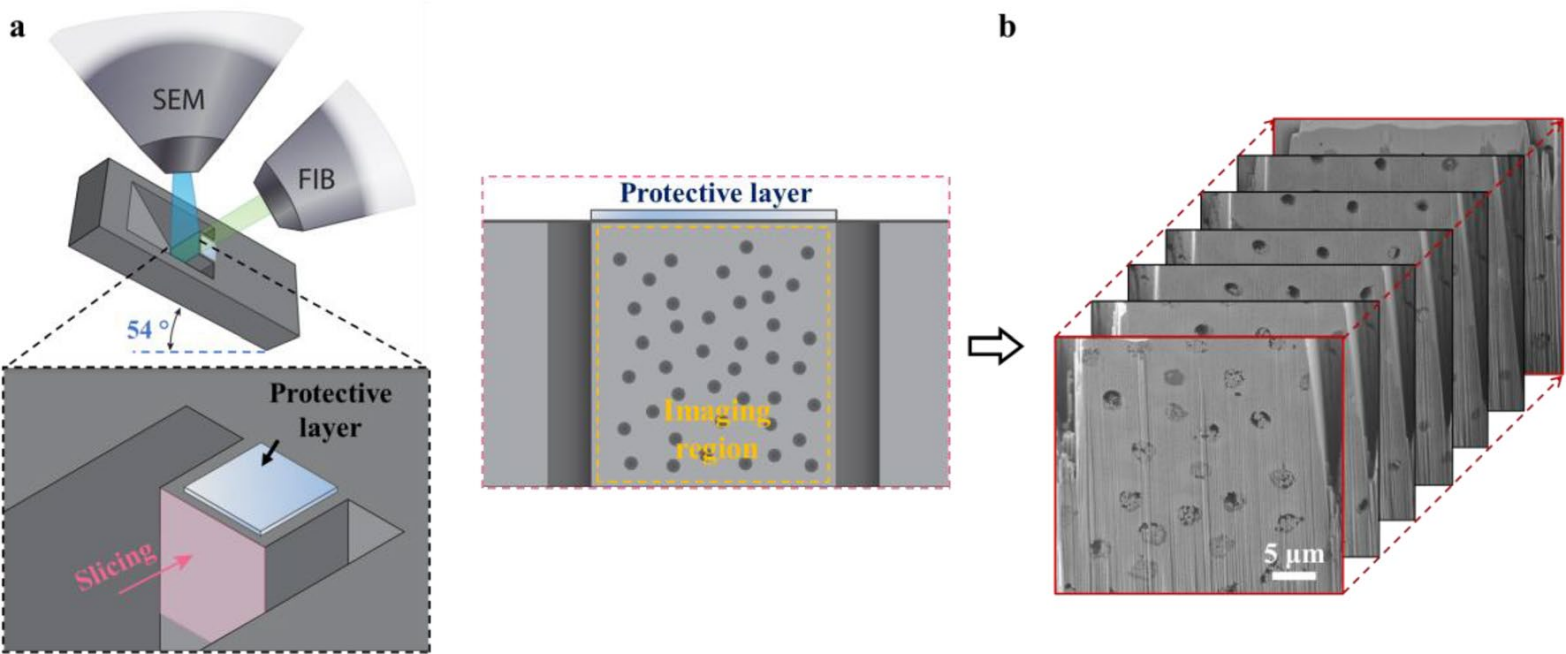
3-D characterization of dentin specimen process using FIB-SEM tomography. (**a**) The schematic of the specimen setup in the FIB-SEM chamber for data acquisition. The pink arrow indicates FIB slicing by the transverse section. The cross-sectional image shows the region of interest for FIB milling and SEM recording. (**b**) 2-D image stacking obtained at different depths along the length direction of the tubules.

To further investigate the efficacy of the test toothpaste for dentin occlusion, specifically, to illustrate dentin occlusion from 3-D domain, FIB-SEM tomography was performed. Figure 3 illustrates the procedure for acquiring the 2-D SEM image stacks used for 3-D reconstruction. As shown in Fig. 3a, the dentin disc treated by the test toothpaste was mounted and tilted at an angle of 54° for the process of FIB milling and SEM imaging (Fig. 3a). The region of interest was trimmed as an island and in situ coated with Pt as a protective layer before the sliced milling by FIB for the SEM image stack acquisition. In the process for acquiring the SEM image stacks, a 20 nm thick slice was milled and then an SEM image was taken of the new exposed cross-section. This process was repeated automatically until the desired distance (the length of the tubules) was reached, producing an SEM image stack as shown in Fig. 3b. The image stacks were then reconstructed to a 3-D volume for the visualization of occluded dentinal tubules.

**Figure 4:**
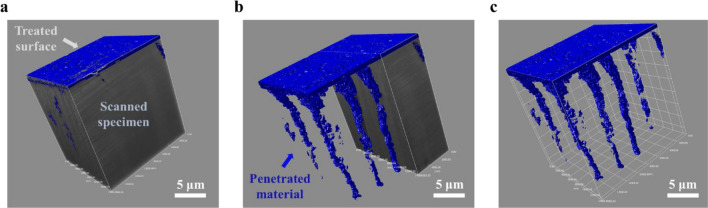
(**a**) The complete specimen consisting of treated surface and scanned dentinal specimen. (**b**) A half-exposed view of scanned specimen detailing the interface between the interior material and the scanned dentin structure. (**c**) The 3-D visualization of exposed plugged material in dentinal tubules. The grid size is 2 μm.

The 3-D reconstruction of the scanned image stacks was accomplished by Dragonfly^@^ software, and a comprehensive visualization of dentin occlusion is illustrated by the video in the Supplementary data. The visualized structure of interest, including the dentinal tubules and the material used for occluding the dentin tubules, is displayed in Fig. 4. Figure 4a and b show a combination of the exposed structure of the material occluded in the tubules and the dentin structure by a digital sectioning function of the software. The material occluded inside the dentin tubules, which may be called a “plug”, can be completely separated from the dentin. As shown in Fig. 4c, the material designed for occluding the dentin tubules, the plugs, occlude almost all of the dentin tubules. The plugs are relatively dense at the top (opening) of the dentin tubules, but are relative loose inside the tubules. These plugs should be able to block fluid from flowing into the dentin tubule and treating dentin hypersensitivity.

**Figure 5:**
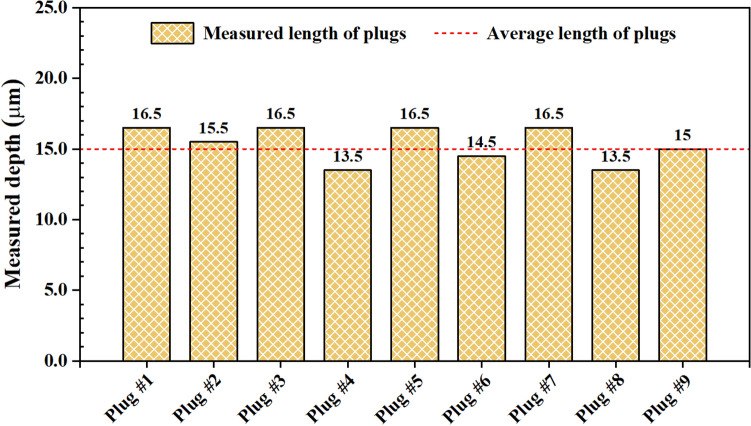
The quantitative measurements of representative plugs from the penetrated tubules by applying the test toothpaste. The average length calculated is 15.3 μm.

The occlusion depth of the dentin disk, which is the length of the plug inside the dentin tubule, was quantitatively measured and plotted as shown in Fig. 5. Nine valid data points were obtained from the 3-D volume, indicating the minimum depth of the occlusion is 13.5 µm and the maximum depth of the occlusion is 16.5 µm. The average occlusion depth of 15.3 µm can be calculated with a standard deviation of 1.3 µm. This measured depth of the plugs can be used to evaluate the efficacy of different toothpastes for anti-hypersensitivity by dentin occlusion.

## Discussion

The occlusion of dentinal tubules has been widely used to treat dentin hypersensitivity by directly interrupting the fluid movement within the dentinal tubules, according to the “hydrodynamic theory” that demonstrates the pain-producing mechanism from the exterior stimulus^26^. In the in vitro study, a versatile method to evaluate the material occluding the tubules is of great importance. Conventional X-ray imaging techniques, such as Computed tomography (CT), 3-D X-ray microcopy, and confocal microscopy, have been used widely to evaluate dentin occlusion in this in vitro study. However, the resolution of these techniques is not high enough to reveal the detailed micro-/nanostructures of most of the material occluding the dentin tubule, as the particle sizes of these material (e.g., silica) are usually in a few tens of nanometers. Although imaging based on synchrotron technology has been claimed with a resolution of 30 nm, the instrument is not readily available. Also, to achieve such a high resolution, the sample thickness needs to be trimmed below 100 µm, which requires FIB milling and extensive preparatory work.

With the advances made in FIB-SEM instrumentation, a very high resolution can be achieved from SEM and a milling thickness of 3 nm can be obtained using FIB. Combining the high-resolution FIB milling and SEM imaging can reveal more significant details between the major tubules of dentin, such as interconnected branching^11^ that includes major branches (0.5–1.0 μm), fine branches (300–700 nm), and microbranches (25–200 nm). These branches can potentially present large amounts of canalicular and anastomosing network. Therefore, study of the intricate intratubular dentin structure is imperative. Serial slices (images) can be automatically obtained and reconstructed by the instrument. By a further imaging process, the structures of interest can be segmented out and displayed individually. In this work, 860 SEM images were obtained with a pixel size of 10 nm × 10 nm and a slice thickness of 20 nm. The images were reconstructed into a 3-D volume, and the material occluded into the dentin tubules were segmented out and demonstrated. The 3-D FIB-SEM tomography can reveal the dentin structure and occluded material with nanoscale resolution, as shown in Fig. 3. The occluded material shows a relatively dense plugging on the top of the dentin tubules. In deeper regions of the tubules, the plugs are not continuous. These detailed structures are directly related the efficacy of the applied toothpaste for treating dentin hypersensitivity by interfering the fluid motion in the dentin tubules.

Compared with the 2-D SEM images, 3-D visualization of dentin occlusion can provide quantitative information of the material occluded in the tubules. As shown Fig. 5, the lengths of the occluding plugs can be quantitatively analyzed from the captured 3-D image. To show the occlusion efficacy of an oral care product or compare the occlusion efficacy of different oral care products, a quantitative analysis is always desired. However, there are not many methods available. This is largely due to the fact that the quantitative analysis requires a high-resolution technique to reveal the nanoparticles in the occluding material. Although X-ray technologies, or the synchrotron technology have been claimed to have the resolution of sub-microns or a few tens of nanometers^16,17^, they still cannot match the sub-nanometer resolution offered by SEM. In this work, we show the process for obtaining SEM images with a pixel size of 10 nm × 10 nm and a slice thickness of 20 nm. Visualization of the particles occluding the dentin tubules was achieved, and the obtained images showed that the occluding material is not continuous inside the tubules. These details, which may not be obtained by other technique, provide the direct reference information for developing toothpastes to treat dentin hypersensitivity.

Most of the advanced SEM/FIB systems can achieve a 5 nm × 5 nm pixel size and 5 nm slice thickness for analyzing dentine structure^27^. However, we noticed some challenges when acquiring serial SEM images from dentin structures using the best resolutions. First, when a small pixel size and slice thickness are used, the image acquisition time is significantly increased, taking one or two days to obtain 2000 images. Because the dentin structure is an organic–inorganic composite, the long imaging time will cause severe change and accumulated heat^28^. As a result, the significant drift will occur, and the automatic tracking of the sample drift may be failed, causing the interruption of the image acquisition process. Thus, when the targeted size (volume) of interest is determined, the pixel size and slice thickness should be reasonably increased to minimize the sample drift.

On top of the sample drift during image acquisition, the organic–inorganic composite nature of dentin may also cause it to receive beam damage from the high energy ions of FIB. The high energy beam can burn out the organics, such as collagen fibers from the dentin structure, and leave micro-pores as shown in Fig. 2b-ii. Thus, when interpreting the cross-sectional SEM images of dentin structure obtained by FIB, the micro-pores generated by ion beam damage may need to be considered as artifacts. To reduce the drift and beam damage, a cryogenic stage or a special sample process can be used if a large volume of interest is desired^29^.

The 3-D reconstruction of SEM images has been improved due to the improvement of commercial software packages. However, there are still challenges for automatically segmenting the region of interest (ROI) from the occluded dentin. One such challenge is that, referring to Fig. 4b, there is not significant contrast difference between the occluding material and dentin. Thus, the segmentation has to be manually achieved from each slice. The deep learning process^30,31^ potentially enables to automatically segment the ROI. However, whether the deep learning process may obtain the desired segmentation results is highly dependent on the ROI. For example, the occluding plugs inside the tubules are most likely to be porous. The deep learning process is often unable to differentiate between the part of the pore that lies in the background of the sliced cross-section and the part of the pore wall that is in that sliced cross-section. This results in artifacts that requires extensive corrections.

In summary, a reliable and desirable 3-D characterization method for dentinal occlusion has been reported here to study the penetration and distribution of applied toothpaste into dentin tubules. The 3-D image can provide detailed information about the dentin occlusion. Moreover, this method could be applied for the quantitative measurements of volumetric occlusions within the tubules, or other microstructural parameters such as porosity, diameter of tubules, and the ratio of peritubular dentin. Dentin specimens analyzed by this 3-D structural visualization approach may help elucidate the changes in dentinal blockage of specimens via testing various occlusion-based treatments in advanced dental research. This method will facilitate understanding the fundamental information of the occlusion efficacy of toothpaste, provide guidance for product development, and better communicate with consumers and professionals.

## Materials and methods

All methods were carried out in accordance with relevant guidelines and regulations. All experimental protocols were reviewed and approved by the Comitato Etico Romano Institutional Review Board. The samples were from male and female adults from the ages of 18 through 70 years (inclusive) and all participants signed an informed consent form.

### Dentinal specimen preparation

The human teeth were cut to several cross-sectional slices (thickness: 600 µm) by using a dicing saw (Buehler IsoMet High Speed Pro, Buehler). Then, these disc-shaped dentin specimens were sanded and polished on a polishing grinder (EcoMet III, Buehler) to create the smooth surface. The polished specimens were etched in 1% citric acid solution for a period of 5 min at room temperature under sonication, followed by cleaning them with the deionized (DI) water for 1 min. Toothpaste slurry was then brushed onto the polished surface of the specimen by a small brush and the specimen was washed in DI water. This process was repeated five times to obtain the final specimen.

### Treatment procedure

At least 3 tested specimens were brushed with the toothpaste slurries for 30 s, by using a microbrush. The tested toothpastes were created by mixing phosphate-buffered saline (PBS) and original toothpaste in a 1:3 ratio. After the occlusion procedure, the tested specimens were placed in 30 mL PBS solution for 15 min at room temperature, followed by stirring at 130 rpm for 15 min. Finally, these treated specimens were rinsed and dried for surface analysis and 3-D FIB-SEM tomography.

### FIB-SEM tomography

The Zeiss^@^ Crossbeam 540 workstation was used to characterize the surface of specimens before and after the test toothpaste treatment without metal coating. The accelerating voltage and beam current were optimized and set to 0.5 kV and 100 pA, respectively. To obtain the serial FIB/SEM images (or slice-and-view), the specimen was mounted on a SEM stub using silver paste and coated with a 200 nm thick platinum (Pt) film. The sample was loaded into the chamber, and the stage was tilted at an angle of 54° to allow the ion beam to be perpendicular to the specimen surface. The accelerating voltage and current of the ion beam were 30 kV and 700 pA, respectively. ZEISS Atlas 5 was used to set up the automatic acquisition of the SEM images of the slices (cross-sections) with a slice thickness of 20 nm, and the pixel size was 10 nm × 10 nm. Dragonfly^@^ was further used to process and reconstruct the SEM images to the 3D volume.

## Supplementary Information

Supplementary Video 1.

## Data Availability

The datasets generated during and/or analyzed during the current study are available from the corresponding author on reasonable request.
